# SWELL signalling in adipocytes: can fat 'feel' fat?

**DOI:** 10.1080/21623945.2019.1612223

**Published:** 2019-05-21

**Authors:** Susheel K. Gunasekar, Litao Xie, Rajan Sah

**Affiliations:** Department of Internal Medicine, Cardiovascular Division, Washington University School of Medicine, St. Louis, MO, USA

**Keywords:** Obesity, ion channel, insulin, caveolae, mechano-transduction

## Abstract

Obesity is becoming a global epidemic, predisposing to Type 2 diabetes, cardiovascular disease, fatty liver disease, pulmonary disease, osteoarthritis and cancer. Therefore, understanding the biology of adipocyte expansion in response to overnutrition is critical to devising strategies to treat obesity, and the associated burden of morbidity and mortality. Through exploratory patch-clamp experiments in freshly isolated primary murine and human adipocytes, we recently determined that SWELL1/LRRC8a, a leucine-rich repeat containing transmembrane protein, functionally encoded an ion channel signalling complex (the volume-regulated anion channel, or VRAC) on the adipocyte plasma membrane. The SWELL1-/LRRC8 channel complex activates in response to increases in adipocyte volume and in the context of obesity. SWELL1 is also required for insulin-PI3K-AKT2 signalling to regulate adipocyte growth and systemic glycaemia. This commentary delves further into our working models for the molecular mechanisms of adipocyte SWELL1-mediated VRAC activation, proposed signal transduction mechanisms, and putative impact on adipocyte hypertrophy during caloric excess.

Obesity is a major public health problem, predisposing to high cholesterol, diabetes and hypertension and inflicting health-care costs over 100 billion dollars in the US alone. It is characterized by a tremendous increase in adipose tissue that is in large part due to massive volumetric expansion of the constituent adipocytes [1,2]. This adipocyte expansion has been long associated with metabolic disease and insulin resistance [1,3–6], implicating adipocyte-size dependent regulation of insulin signalling and growth. Studies in cultured adipocytes show that lipid droplet growth increases local tissue stiffness to mechanically-stimulate lipogenesis, and adipocyte growth in neighbouring cells via MEK signalling [7–9], suggesting that obesity may beget obesity in a positive-feedback, feed-forward manner [7]. Indeed, elegant studies in primary, mature, human adipocytes also reveal evidence of adipocyte mechano-signalling in human adipose tissue [10]. Taken together, this literature suggests that, in the setting of obesity, adipocyte expansion may activate an undiscovered mechano-sensitive molecule that modulates adipocyte growth and intracellular signalling. We recently discovered that SWELL1 (LRRC8a), a member of the *L*eucine *R*ich *R*epeat *C*ontaining protein family, is a required component of a volume-sensitive and putatively mechano-sensitive ion channel/signalling molecule that is activated in the setting of adipocyte hypertrophy and regulates adipocyte size, insulin signalling and systemic glycaemia via a novel SWELL1-PI3K-AKT2-GLUT4 signalling axis. Adipocyte-specific *SWELL1* ablation disrupts insulin-PI3K-AKT2 signalling, inducing insulin resistance and glucose intolerance in *vivo* [11]. These data identify SWELL1 as a component of a cell-autonomous sensor of adipocyte size and a *positive* regulator of adipocyte insulin signalling, growth and glucose homeostasis, particularly in the setting of obesity (Figure 1). Thus, SWELL1 may be a component of the molecular mechano-sensor responsible for the mechano-sensitive nature of adipocytes described by others previously [7–10].

**Figure 1.**
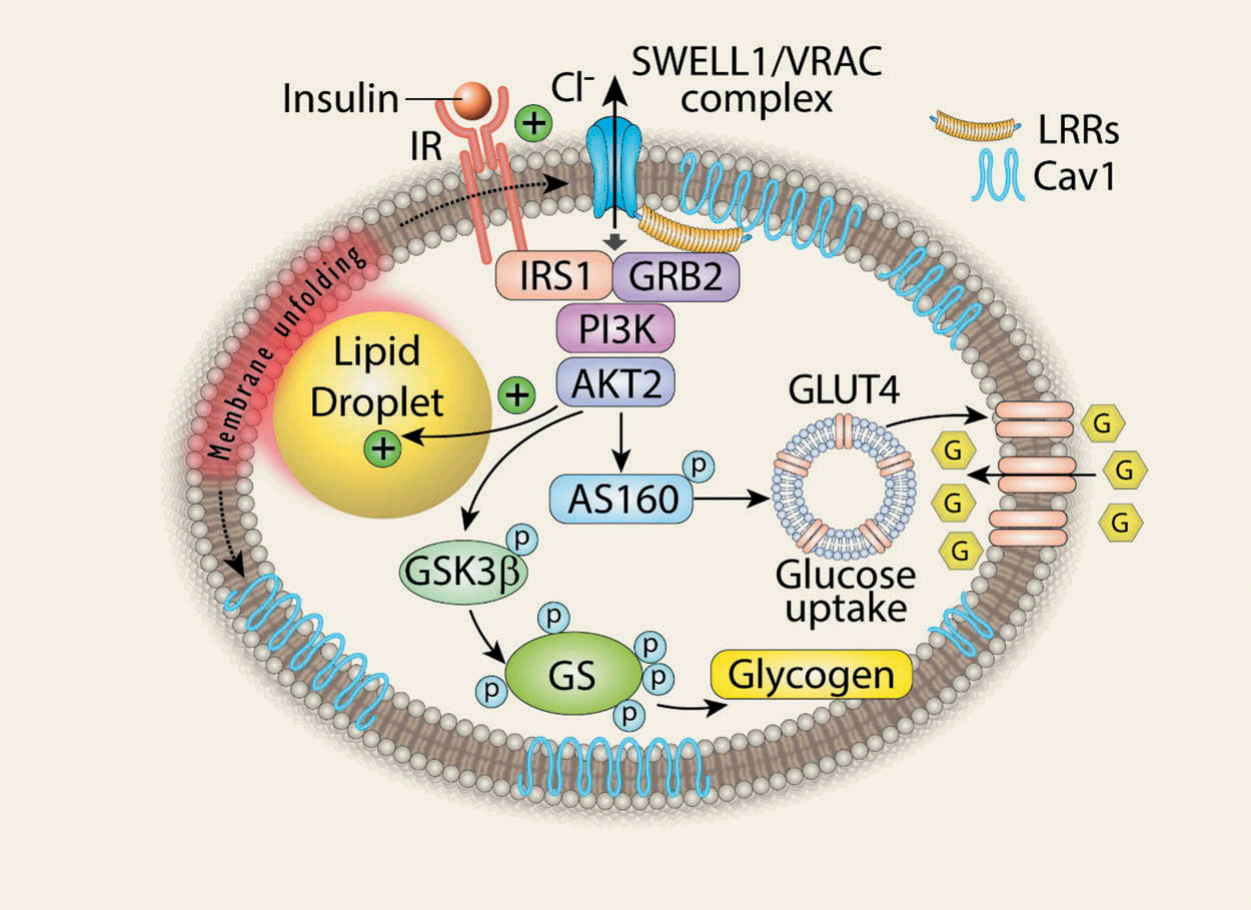
Adipocyte SWELL1 is a positive regulator of insulin signalling and glucose homeostasis. LRR: Leucine Rich Repeat; Cav1: Caveolin-1; GSK3β: Glycogen Synthase 3β; GS: Glycogen Synthase. (Zhang Y. et al., *Nature Cell Biology* 2017).

## SWELL1/LRRC8a, a macromolecular mechano-sensitive complex?

**Figure 2.**
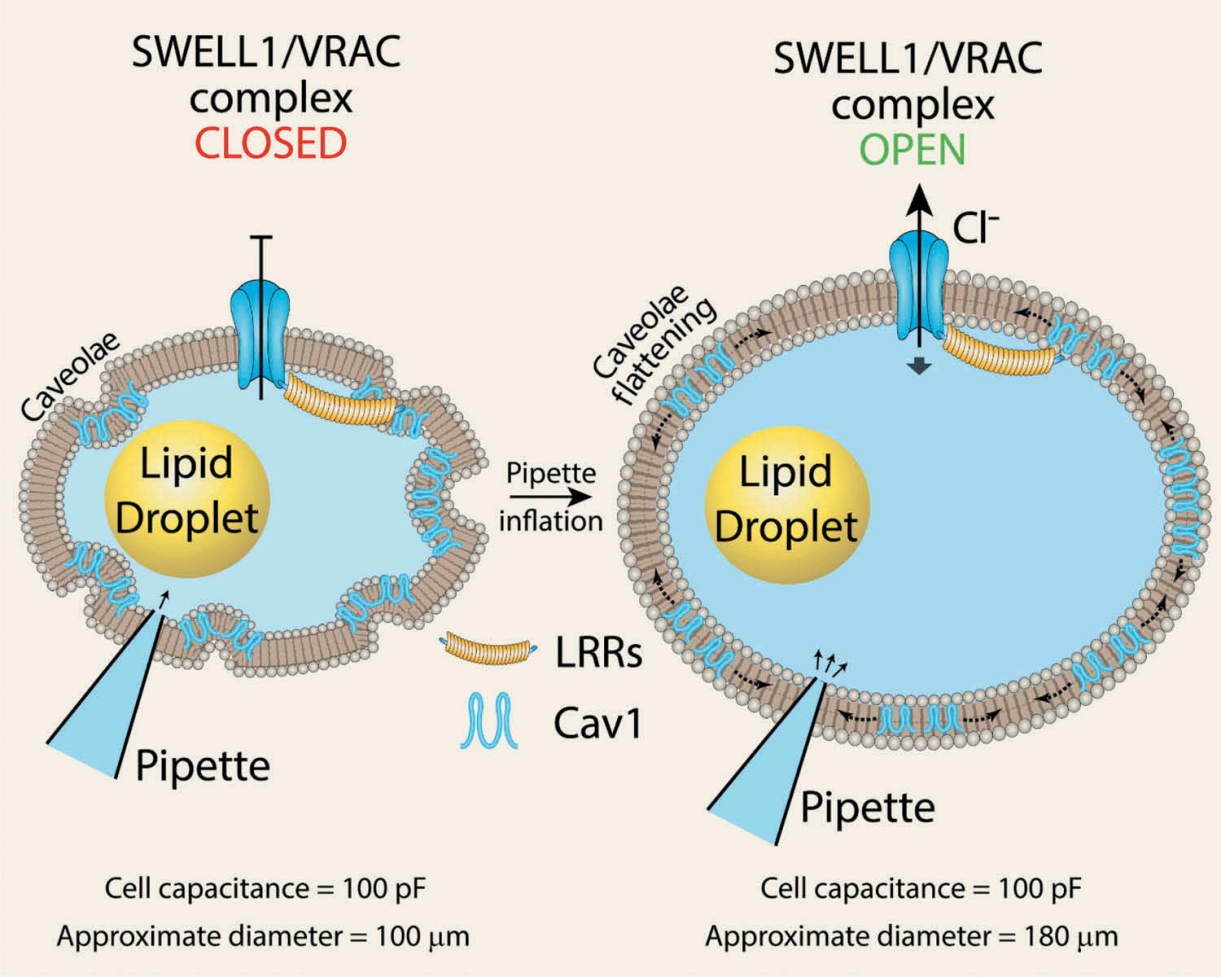
SWELL1-mediated VRAC activation by mechanical adipocyte swelling. Mechanical swelling increases adipocyte size without a measurable change in total surface area (as assessed by total cell capacitance) implicating caveolae flattening as a mechanism of VRAC activation in adipocytes. LRR: Leucine Rich Repeat; Cav1: Caveolin-1.

While it is now established that SWELL1 functionally encodes the volume-regulated anion channel (VRAC) or the swell-activated chloride current (I_Cl,SWELL_) in multiple cell types, such as common cell lines [12,13], adipocytes [11], pancreatic β-cells [14,15], nodose neurons [16], astrocytes [17] and spermatozoa [18], it remains unclear whether SWELL1-mediated VRAC is mechano-sensitive, responsive to osmolarity, ionic strength, or other stimuli [19]. Recent work using purified LRRC8 proteins reconstituted in lipid droplets demonstrates that VRAC channels are only activated by substantial, rather non-physiological reductions in ionic strength, and not mechanical or osmotic stimuli [20]. These findings imply that the physiological function of VRAC is to sense ionic strength. However, there are a number of papers, spanning over a decade, that demonstrate VRAC activation in response to purely mechanical forces, such as pulling on β1-integrins with magnetic microparticles in cardiac myocytes [21,22] and with shear force or positive pressure inflation in endothelial cells [23–26] (for excellent reviews see [19,27]). A key difference in these studies is measuring VRAC activity in an intact cell versus in a minimalist system that is devoid of the requisite interacting proteins necessary for SWELL1 to function within a physiological cellular context. However, this is not to say that VRAC is not, or cannot, be activated by prominent reductions in ionic strength in intact cells as this has also been shown very elegantly in endothelial cells [28]. Zhang et al. (2017) demonstrated that SWELL1-mediated VRAC activates in response to positive pressure ‘inflation’ with pipette solution (with as little as 1 mmHg pressure) after achieving whole-cell configuration [11,29]. Under these conditions, both ionic and osmotic gradients equilibrate within a minute of cell dialysis with pipette solution and remain constant thereafter. The subsequent VRAC activation observed upon mechanical adipocyte swelling must, therefore, occur independent from changes in ionic or osmotic strength. It is notable that whole-cell adipocyte capacitance (which is a measure of total surface area) prior to mechanical swelling is identical to the capacitance measured after swelling. This indicates that the highly caveolated adipocyte plasma membrane (PM), enriched in caveolin-1, is unfolding upon positive pressure swelling (Figure 2). We surmise that this PM unfolding and caveolae flattening may form part of a mechano-sensitive activation mechanism for SWELL1-mediated VRAC (Figure 2). In support of this hypothesis, there is prior evidence that caveolae are required for VRAC activation [30–32], that caveolae provide ‘membrane reserves’ to regulate VRAC activation [33], and that caveolae are mechano-sensors that flatten into the plasma membrane in response to mechanical stimuli [34,35]. Moreover, similar to SWELL1, caveolin-1 regulates insulin-PI3K-AKT signalling [36–41] and adipocyte lipid content [42–44]. Indeed, we showed that Cav1 and SWELL1 interact, probably via GRB2, in microdomains in the adipocyte membrane [11]. Curiously, endothelial cells, like adipocytes, are highly enriched in caveolae, and exhibit mechano-activatable VRAC [25], suggesting the presence of a similar SWELL1-GRB2-Cav1 mechano-sensitive macromolecular complex in the endothelium as well. Taken together, these data support the concept that purified LRRC8 channels are not intrinsically mechano-sensitive when reconstituted in a minimalist system. However, in a native cellular environment SWELL1/LRRC8a form macromolecular complexes with interacting proteins (GRB2, Cav1, and possibly cytoskeletal proteins, integrins) that confer mechano-sensitivity [19] and mechano-signalling properties to regulate insulin-AKT2 signalling, growth, and possibly other signalling pathways as well.

## SWELL1/LRRC8a: channel, scaffolding protein, insulin signalling rheostat

**Figure 3.**
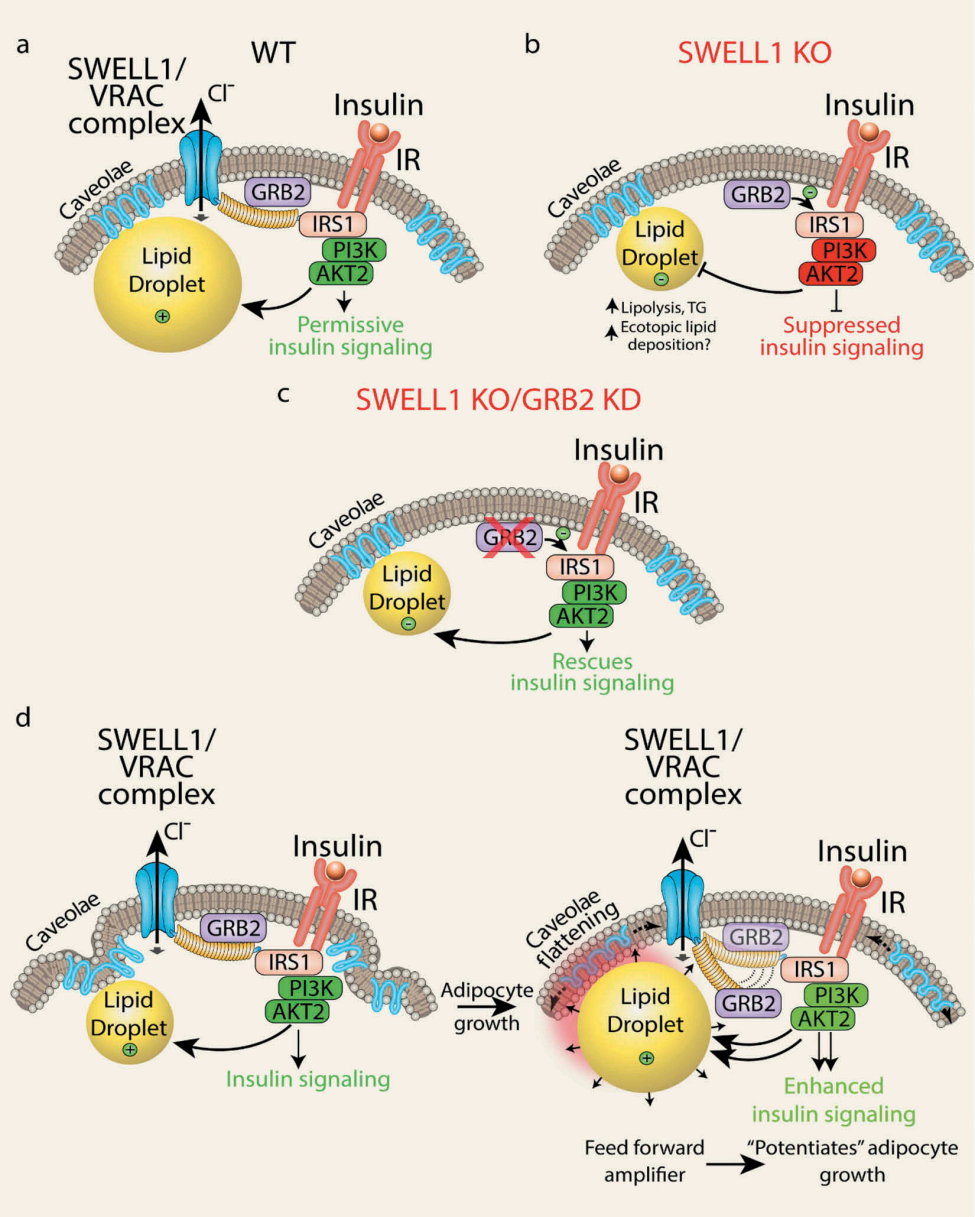
Adipocyte SWELL1/VRAC complex regulates insulin-GRB2-PI3K-AKT2 signalling and senses mechanical forces via caveolae flattening to tune insulin-AKT2 signalling by modulating GRB2-IRS1 interactions. **(a)** In WT adipocytes, SWELL1 restrains GRB2 from suppression of PI3K-AKT2 signalling. **(b)** SWELL1 ablation releases GRB2 to fully inhibit insulin-PI3K-AKT2 signalling. **(c)** GRB2 knock-down (KD) in SWELL1 KO adipocytes fully rescues insulin-PI3K-AKT2 signalling. **(d)** Working model: adipocyte expansion activates the SWELL1/VRAC complex to disinhibit insulin-PI3K-AKT2 via GRB2, and thereby support lipogenesis and continued adipocyte growth, in a feed-forward manner, potentiating adipocyte hypertrophy.

Historically, VRAC has been studied for decades using hypotonic swelling as the primary stimulus for VRAC activation and for observing regulatory volume decrease (RVD) [45]. Accordingly, the primary function ascribed to VRAC, for which it was ultimately named, was to regulate cytoplasmic volume in response to hypotonic swelling. However, SWELL1 is broadly expressed, and VRAC also ubiquitously present in cell types that are never exposed to a hypotonic environment, nor to significant drops intracellular ionic strength – i.e. adipocytes, pancreatic β-cells, endothelial cells. Therefore, the true physiological function of the SWELL1 channel complex may have little to do with cellular cytoplasmic volume regulation under physiological conditions in most mammalian cells. The SWELL1 loss-of-function experiments presented in Zhang et al. instead reveal a signalling mechanism in which the SWELL1 complex, via the C-terminal leucine-rich repeat domain (LRRD), regulates insulin-PI3K-AKT2 signalling by titrating the interaction of a negative regulator, GRB2, with the insulin signalling pathway (IRS1). The working model based on these data, is that SWELL1 physically restrains GRB2 from inhibiting AKT2 signalling, in an insulin-dependent manner (Figure 3(a)). In the absence of SWELL1, GRB2 is free to fully suppress insulin-AKT2 signalling (Figure 3(b)). Consistent with this model, selective GRB2 ablation can fully rescue insulin-AKT2 signalling in SWELL1 KO adipocytes (Figure 3(c)) [11].

Outstanding questions are whether: 1. SWELL1/LRRC8 complex functions merely as a scaffolding protein for components of the insulin signalling transduction machinery, since LRRD are known to provide surfaces for protein–protein interactions [46–49]; or 2. SWELL1 forms a mechano-sensitive, macromolecular complex that senses cell expansion via conformational changes stimulated by caveolae flattening, etc. (as evidenced by current activation) and then transduce GRB2-dependent modulation of insulin signalling by physically destabilizing the GRB2-IRS1 interaction – thereby ‘tuning’ insulin-AKT2 signalling in response to adipocyte growth. This may establish a feed-forward mechanism to potentiate adipocyte growth during times of nutrient excess (Figure 3(d)). Although scenario #2 is inferred by Zhang et al., these studies largely focused on SWELL1 loss-of-function experiments *in vitro* and *in vivo*. In animal studies, we used high-fat diet models to induce physiological adipocyte expansion and presumably activate SWELL1-mediated signalling. However, these approaches do not allow for tracking SWELL1 signalling in real-time during adipocyte growth, and the hypothesized unfolding of the highly caveolated adipocyte plasma membrane as proposed in our working model (Figure 3(d)) – thus these specific models must be formally tested. Indeed, a prediction of this model is that induction of SWELL1 protein expression, SWELL1-LRRC8 activity and VRAC current is a feature of early, normoglycemic obesity that supports healthy adipocyte expansion and maintains insulin-sensitivity [11,50]. Consequently, reduced SWELL1 activity and associated VRAC currents in the setting of later stage, hyperglycaemic obesity may contribute to insulin resistance and exacerbate impairments in systemic glycaemia, as shown by Xie et al. (2017) [50]. Consistent with this hypothesis, Inoue et al. (2010) [51], the first group to measure VRAC in freshly isolated mature white adipocytes, showed that VRAC is indeed significantly reduced 2–3 fold in the KKA^y^ polygenic Type 2 diabetes model compared to either control KKA^a^ or C57 mice. These data lend further support to the notion that SWELL1 is permissive for insulin-signalling [11,14], potentially bridges adipocyte expansion with the regulation of insulin-AKT2 signalling [7–11], and may become deficient in T2D states to drive diabetes pathogenesis [50,51]. From an overarching cell biology perspective, this model is physiologically relevant in a multitude of cell types and tissues whereby the SWELL1 complex may integrate cellular growth or mechanical force inputs to tune insulin-AKT or mTOR signalling, including endothelium, cardiac muscle, skeletal muscle, and macrophages to name just a few. Finally, a critical unanswered question that arises from the above working model is whether SWELL1-mediated regulation of insulin-AKT2 signalling is mediated purely via SWELL1-dependent GRB2-IRS1 protein–protein interactions or if SWELL1-LRRC8 channel activity (osmolite or chloride conductance) also contributes. Indeed, this is an area of ongoing investigation in the laboratory and has been recently facilitated by a deluge of novel cryo-electron microscopy data of LRRC8a and LRRC8a/c hexamers within the past year [52–55].

In summary, SWELL1 is a component of a novel, broadly expressed, ion channel signalling complex that we hypothesize senses adipocyte growth, putatively via plasma membrane and caveolae unfolding mechanisms to regulate insulin-PI3K-AKT2 signalling and glucose metabolism. Future studies of this fascinating signalling molecule will broaden our mechanistic understanding of its endogenous physiological activation mechanisms, intracellular localization, interaction partners, and regulation of intracellular signalling and systemic energy homeostasis.
